# Antioxidant activities and oxidative stress inhibitory effects of ethanol extracts from *Cornus officinalis* on raw 264.7 cells

**DOI:** 10.1186/s12906-016-1172-3

**Published:** 2016-07-08

**Authors:** Kyung-A Hwang, Yu-Jin Hwang, Jin Song

**Affiliations:** Department of Agrofood Resources, National Academy of Agricultural Science, Rural Development Administration, Wanju-gun, Jeollabuk-do 55365 Republic of Korea

**Keywords:** *Cornus officinalis*, Oxidative stress, Antioxidant activity

## Abstract

**Background:**

*Cornus officinalis*, is a deciduous tree native to the eastern Asia, distributes mainly in (e.g. Korea, as well as China, and Japan). It is used as folk medicine to backache, polyuria, hypertension and nervous breakdown. Pharmacological studies have demonstrated that *C. officinalis* possess anti-oxidant, anti-hyperglycemic, and immune regulatory effects. However, reports on the antioxidant activity of *C. officinalis* have been limited to *in vitro* radical scavenging studies. Its mechanism of action within the cell at the genetic level especially has not yet been clearly defined. Therefore, we investigated the anti-antioxidant activities of *C. officinalis* in RAW 264.7 cells.

**Methods:**

The antioxidant activities and protective effects of *C. officinalis* ethanol extract on cell damage and the antioxidant enzyme system in lipopolysaccharide (LPS)-induced oxidative stress-damaged RAW 264.7 cells were assessed. To measure the effects of *C. officinalis* on antioxidant activities, we used the following methods: Total phenol and flavonoid contents, DPPH scavenging activity assay, ABTS scavenging activity assay, FRAP value measurement, xanthine oxidase activity assay, ROS generation measurement and real time PCR.

**Results:**

The total phenol and flavonoid contents of *C. officinalis* extracts were 27.04 mg GAE/g and 3.70 mg QE/g, respectively. The antioxidant activities of *C. officinalis* extracts increased in a dose-dependent manner: the IC_50_ values for DPPH and ABTS radical scavenging activities of *C. officinalis* extracts were 99.32 μg/mL and 138.51 μg/mL, respectively. *C. officinalis* extracts inhibited xanthine oxidase activity and reactive oxygen species generation. The expression of antioxidant enzymes, Cu/ZnSOD, MnSOD, catalase, and glutathione peroxidase increased upon treatment with *C. officinalis* extracts at 100 μg/mL, compared to that in the LPS-treated group.

**Conclusions:**

These results suggest the therapeutic potential of *C. officinalis* extract as an anti-oxidant agent.

**Electronic supplementary material:**

The online version of this article (doi:10.1186/s12906-016-1172-3) contains supplementary material, which is available to authorized users.

## Background

Reactive oxygen species (ROS) are produced in oxidation processes essential to most living organisms and are essential to produce the energy required to fuel other biological processes. However, excessive production of ROS is damaging to cells because ROS destroy molecules such as DNA and proteins. Thus, ROS play an important role in the pathogenesis of various serious diseases, such as neurodegenerative disorders, cancer, cardiovascular diseases, atherosclerosis, cataracts, and inflammation [1–5]. The mechanism of inflammation injury partially involves the release of ROS from activated neutrophils and macrophages. ROS propagate inflammation by stimulating the release of cytokines such as interleukin-1, tumor necrosis factor-α, and interferon-α, which stimulate recruitment of additional neutrophils and macrophages. Free radicals are important mediators that provoke or sustain inflammatory processes, and consequently, their neutralization by antioxidants and radical scavengers can attenuate inflammation [6, 7]. Therefore, compounds that have scavenging activities toward these radicals could have therapeutic potential.

There are two methods of suppressing ROS. First, natural defense mechanisms present in the human/mammalian system counteract the potential deleterious effects of ROS. Normally, cells have antioxidant systems that protect against the harmful effects of ROS, including superoxide dismutase (SOD), which converts superoxide anions to hydrogen peroxide (H_2_O_2_) for rapid removal by detoxifying enzymes such as glutathione peroxidase (GPx). Similarly, glutathione (GSH) can reduce ROS for GPx-catalyzed H_2_O_2_ reductions [8, 9]. Second, functional components from the external environment such as flavonoids, L-ascorbic acid (Vit C), and α-tocopherol (Vit E), act as antioxidants [10, 11]. Therefore, a diet rich in antioxidants could help the body defend itself against the molecular effects of free radicals and ROS and hinder the development of many chronic diseases. Numerous studies have shown that natural dietary compounds can potently modulate oxidative stress. Plant phenolics, flavonoids, tannins, and anthocyanins have useful properties such as antioxidant, immune, and anticancer activities. The presence of these antioxidants increases the efficacy of the protection system against ROS [12–14].

*Cornus officinalis* (Cornaceae) is a deciduous tree native to eastern Asia (e.g., Korea, China, and Japan). The fruit of *C. officinalis*, known as “Sansuyu” in Korean, is mainly harvested in the central and southern regions of Korea [15]. *C. officinalis* is often included in traditional treatments for conditions such as backache, polyuria, hypertension, and nervous breakdown [16]. Pharmacological studies have demonstrated that *C. officinalis* possesses antioxidant [17], antihyperglycemic [18], immune regulatory [19] and anti-inflammatory effects [20].

Furthermore, many functional compounds such as ursolic acid, tartaric acid, malic acid, glucosides, and fatty acids are present in the fruit. Several studies have also reported that these compounds have antioxidant and anti-inflammatory effects [21–24].

Reports on the antioxidant activity of *C. officinalis* have been restricted to *in vitro* radical scavenging studies. Its mechanism of action within the cell at the genetic level has not yet been clearly defined. Therefore, the aims of this study were to identify the effect of *C. officinalis* on antioxidant activity, inhibition of ROS production, and antioxidant-related gene expression in RAW 264.7 cells (murine macrophage cell line). This study suggests that the ethanol extract of *C. officinalis* could be used as a natural source of antioxidants in the food and pharmaceutical industries.

## Methods

### Reagents

Folin-Denis reagent, sodium carbonate, aluminum chloride, potassium acetate, potassium persulfate, 1,1-diphenyl-1-picrylhydrazyl (DPPH), 2,2′-azinibis 3-ethyl benzothiazoline-6-sulfonic acid (ABTS), 2,4,6-tris(2-pyridyl)-s-triazine (TPTZ), iron(III) chloride hexahydrate, gallic acid, acetic acid, lipopolysaccharide (LPS), 3-(4,5-dimethylthiazol-2-yl)-2,5-diphenyltetrazolium bromide (MTT), ascorbic acid (Vit C), and quercetin were purchased from Sigma-Aldrich (St. Louis, MO, USA). Iron (II) sulfate heptahydrate (FeSO_4_) was purchased from Junsei (Tokyo, Japan). Dulbecco’s Modified Eagle’s Medium (DMEM), fetal bovine serum (FBS), phosphate-buffered saline (PBS), penicillin-streptomycin (P/S), and trypsin-EDTA were obtained from Gibco (Waltham, MA, USA). The xanthine oxidase (XO) assay kit was purchased from Abcam (Cambridge, MA, USA). The other reagents used were of analytical grade.

### Sample preparation and extraction

*C. officianalis* was purchased from Korea Medicine Herbal Association, which is under the jurisdiction of the Ministry of Agriculture, Food and Rural Affairs (Seoul, Korea). The plant was identified and authenticated by the Korea Medicine Herbal Association. Voucher specimens (NAAS-15-03) were deposited at the Department of Agrofood Resources Herbarium, National Academy of Agricultural Science, Korea. *C. officianalis* (20 g) was extracted twice with 70 % ethanol at 70 °C for 6 h. The 70 % ethanol extract was filtered using filter paper (Advantec, Tokyo, Japan). Subsequently, the filtrates were combined and evaporated under vacuum (EYELA CCA-1110, Tokyo Rikakikai Co., Tokyo, Japan) and then lyophilized with a freeze dryer (Ilshine Lab, Suwon, Korea) at −70 °C under reduced pressure (<20 Pa). The dry residue was stored at −70 °C. For further analysis, the dry extract was reconstituted with dimethyl sulfoxide (DMSO).

### Total phenolic content

The total phenol content of *C. officianalis* extract was determined by the Folin-Ciocalteau method [25]. The extract was oxidized with Folin-Ciocalteau reagents, and then the reaction was neutralized with saturated sodium carbonate. After incubation at room temperature for 1 h, the absorbance of the reaction mixture was measured at 725 nm using a microplate reader (Molecular Devices, Sunnyvale, CA, USA). The total phenolic content is expressed as gallic acid equivalents in milligrams per gram (mg GAE/g) of dry extract.

### Total flavonoid content

A sample solution was mixed with 100 % ethanol, 10 % aluminum chloride, 1 M potassium acetate, and distilled water. The reagents were thoroughly mixed and allowed to stand for 40 min at room temperature, and the absorbance of the supernatant was measured at 415 nm [26]. Quercetin was used to plot a standard calibration curve, and the results are expressed as quercetin equivalents in milligram per gram (mg QE/g) of dried extract.

### DPPH radical-scavenging activity

The DPPH radical-scavenging activity was carried out according to the Blois method [27]. DPPH (0.3 mM) was added to each sample. After incubation for 30 min in the dark at room temperature, the absorbance was measured at 518 nm using a microplate reader. Vit C was used as a positive control. The free radical-scavenging capacity was expressed by IC_50_.

### ABTS radical cation-scavenging activity

The ABTS assay was performed to evaluate the ability of the *C. officianalis* extract to scavenge the ABTS radical cation in comparison to that of a standard (Vit C) [28]. The radical cation was prepared by mixing 7 mM ABTS with 2.45 mM potassium persulfate (1:1 v/v) and leaving the mixture for 24 h until the reaction was completed and the absorbance was stable. The ABTS radical solution was diluted with PBS to an absorbance of 0.7 (±0.02) at 732 nm. The assay was conducted with diluted ABTS radical solution mixed with samples, and the measurements were taken at 734 nm after 30 min. The antioxidative activity of the samples was calculated by determining the decrease in absorbance. The free radical-scavenging capacity was expressed by IC_50_.

### Ferric-reducing antioxidant power (FRAP) activity

FRAP activity was determined using manual assay methods [29]. The working fluid was freshly prepared by mixing acetate buffer (300 mM, pH 3.6) with 10 mM TPTZ in HCl and 20 mM iron (III) chloride hexahydrate. The sample solution or Vit C was added to working fluid, and the mixture was left for 4 min at room temperature. The absorbance was measured at 593 nm. The results are expressed as FeSO_4_ equivalents.

### Cells and culture

RAW 264.7 cell lines were purchased from the Korean Cell Line Bank (Seoul, Korea). The cell lines were grown in DMEM with 10 % FBS and 1 % P/S, and incubated at 37 °C in 5 % CO_2_.

### Cell cytotoxicity assay

RAW 264.7 cells were plated at 1 × 10^4^ cells/well. The *C. officianalis* ethanol extract in DMSO was diluted in PBS to obtain final concentrations of 10, 50, and 100 μg/mL. Cells were treated with samples for 24 h and MTT solution was added. After 4 h, the media were removed and DMSO was added to each well. The resulting absorbance was measured at 540 nm.

### Xanthine oxidase inhibitory activity assay

XO inhibitory activity was assayed using a commercial xanthine oxidase assay kit (Abcam, Cambridge, MA, USA) according to the manufacturer’s instructions. Briefly, RAW 264.7 cells were plated at 5 × 10^5^ cells/well. After 4 h, the cells were treated with 10, 50, and 100 μg/mL of *C. officianalis* for 24 h. The treated cell pellets were mixed with assay buffer and the supernatants were isolated. The working solutions were added to the supernatants and incubated at 37 °C. After 1 h, the absorbance was measured at 570 nm.

### Intracellular reactive oxygen species scavenging activity

RAW 264.7 cells were plated at 1 × 10^6^ cells/well. After 4 h, the cells were treated with 10, 50, and 100 μg/mL *C. officianalis* and LPS for 24 h. After incubation, the cells were washed with PBS and harvested. The cells were then incubated with dichlorofluorescein diacetate (DCF-DA) (25 μM) for 30 min at 37 °C in the dark. After several washings with PBS, the fluorescence was captured using a FACSCalibur flow cytometer (BD Biosciences, San Jose, CA, USA). DCF fluorescence was measured at an excitation wavelength of 488 nm and emission wavelength of 515–540 nm.

### Real-time reverse transcription polymerase chain analysis

To determine the expression levels of Cu/Zn SOD, Mn SOD, catalase, and GPx, real-time reverse transcription polymerase chain reaction (RT-PCR) was performed using a real-time thermal cycler Qiagen Rotorgene Q (Valencia, CA, USA), in accordance with the manufacturer’s instructions. The cells were treated with *C. officianalis* extracts and cultured for 24 h. Thereafter, cDNA was synthesized from the total RNA isolated from cells. The PCR reaction was performed using the 2X SYBR Green mix (Qiagen, Valencia, CA, USA). All results were normalized to glyceraldehyde 3-phosphate dehydrogenase (GAPDH) expression. The following primer sequences were used for real-time RT-PCR: GAPDH, 5′-GAG CCA AAA GGG TCA TCA TC-3′ (forward), 5′-TAA GCA GTT GGT GGT GCA GG-3′ (reverse); Cu/Zn SOD, 5′-CAG CAT GGG TTC CAC GTC CA-3′ (forward), 5′-CAC ATT GGC CAC ACC GTC CT-3′ (reverse); Mn SOD, 5′-GGG TTG GCT TGG TTT CAA TAA GGA A-3′ (forward), 5′-AGG TAG TAA GCG TGC TCC CAC ACA T-3′ (reverse); catalase, 5′-AAG ACA ATG TCA CTC AGG TGC GGA-3′ (forward), 5′-GGC AAT GTT CTC ACA CAG GCG TTT-3′ (reverse); and GPx, 5′-CTC GGT TTC CCG TGC AAT CAG-3′ (forward), 5′-GTG CAG CCA GTA ATC ACC AAG-3′ (reverse).

### Statistical analysis

Statistical analysis was performed using SPSS (version 17.0; SPSS Inc., Chicago, IL, USA). Descriptive statistics were used to calculate the mean and standard error of the mean (SEM). One-way analysis of variance was performed, and when significance (*p* < 0.05) was found, the differences of mean values were identified with Duncan’s multiple range tests.

## Results and Discussion

### Total phenolic and flavonoid contents

The total phenolic and flavonoid contents of the *C. officianalis* extract were determined through a linear gallic acid and quercetin standard curve and are expressed as mg GAE/g and mg QE/g, respectively. As shown Table 1, the total phenolic and flavonoid contents of *C. officianalis* were 27.04 ± 0.61 mg GAE/g and 3.70 ± 0.08 mg QE/g, respectively. According to Jeon et al. [30], the total phenolic and flavonoid contents of *C. officianalis* ethanol extract were 34.22 mg/g and 5.67 mg/g, respectively, similar to our findings.

**Table 1:** Total phenolic and flavonoids contents of *Cornus officinalis* ethanol extract

Sample	Total polyphenol^a^ (mg GAE/g)	Total flavonoid^b^ (mg QE/g)
*Cornus officinalis*	27.04 ± 0.61	3.70 ± 0.08

^a^Total phenolic content expressed in mg of gallic acid equivalent (GAE) per gram of dry weight of plants

^b^Total flavonoid content expressed in mg of quercetin equivalent (QE) per gram of dry weight of plants

Phenolic compounds have been reported to be associated with antioxidant activity, anticancer effects, and other biological functions, and may prevent the development of aging and disease [31–33]. Thus, our study results suggest that *C. officianalis* extracts might have high antioxidant activities.

### Antioxidant capacities of *C. officianalis*

ROS scavenging has been reported to be a very important antioxidant mechanism in inhibiting lipid oxidation and aging in the human body [34].

**Fig. 1:**
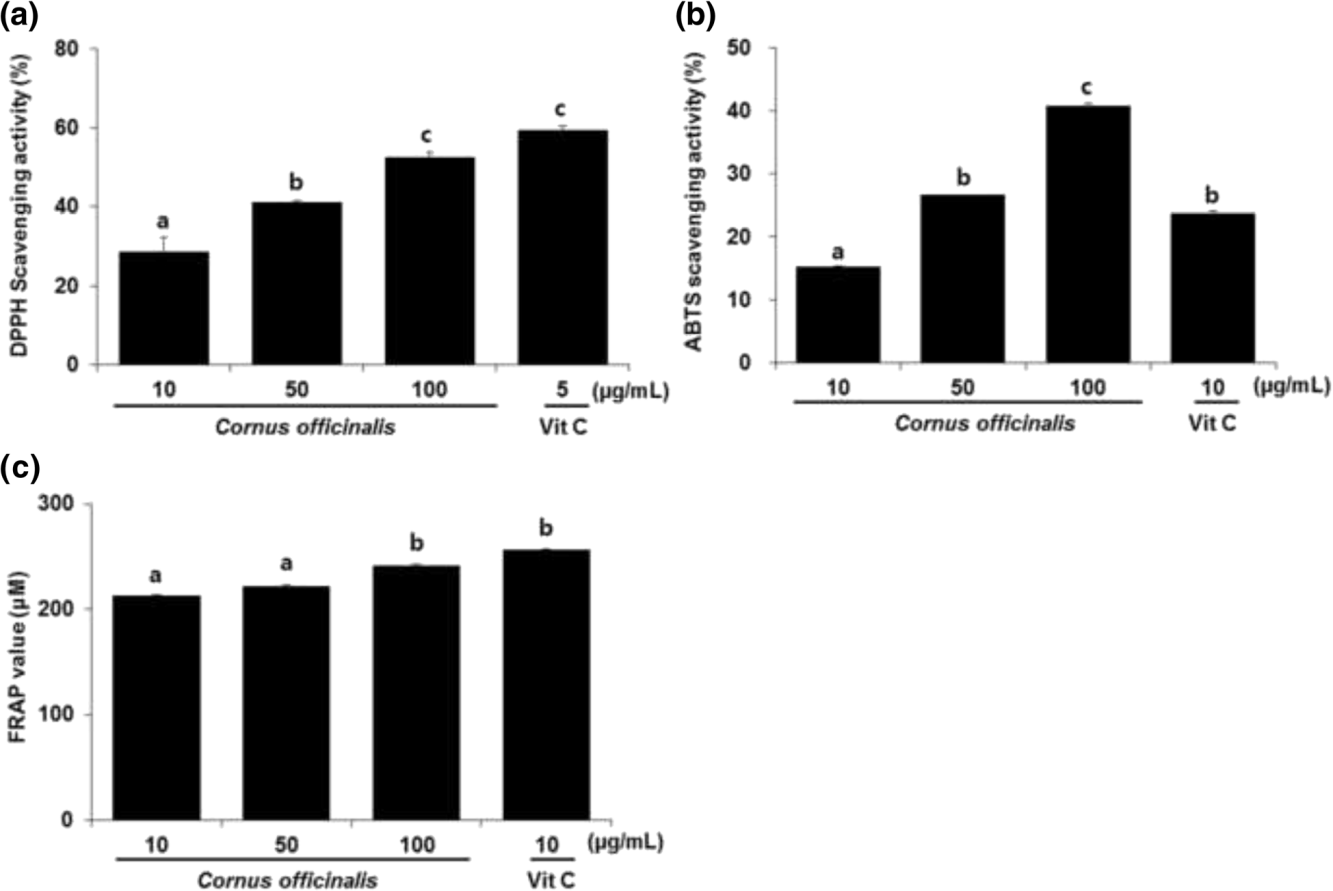
Antioxidant activities of various concentrations of *Cornus officinalis*. **a** DPPH scavenging activity. **b** ABTS radical scavenging activity. **c** FRAP value. Values are the mean ± SEM of experiments in triplicate (*n* = 3). Values expressed by different letters are significantly different at *p* < 0.05

The radical scavenging capacities, as determined by DPPH, ABTS and FRAP assays, are shown in Fig. 1. Upon interacting with DPPH, antioxidants transfer either an electron or a hydrogen atom to DPPH, thus neutralizing its free radical character. Therefore, absorption at 518 nm is proportional to the amount of residual DPPH [35]. The radical scavenging activity was found to be 28.4 %, 40.9 %, and 52.44 %, at 10, 50, and 100 μg/mL of *C. officianalis* ethanol extract (IC_50_ = 99.32 μg/mL), respectively. Our results (IC_50_ = 99.32 μg/mL) were higher than those obtained by Jeon et al. (IC_50_ = 154 μg/mL) [31]. DPPH radical scavenging activity is hypothesized to depend on the growth and extraction conditions of *C. officinalis.*

The ABTS assay measures the relative ability of an antioxidant to scavenge the ABTS generated in the aqueous phase by reacting a strong oxidizing agent (potassium persulfate) with ABTS salt. Reduction of the blue-green ABTS radical solution is measured [29]. The *C. officianalis* ethanol extract was shown to have scavenging activity in a dose-dependent manner; the scavenging activity was 40.7 % after exposure to 100 μg/mL of *C. officianalis.*

The FRAP assay measures total antioxidant activity via the reduction of the ferric tripyridyltriazine (Fe^3+^-TPTZ) complex to the ferrous form [30]. The ferric complex-reducing ability of *C. officianalis* was similar to the results obtained for the radical scavenging assay. *C. officianalis* (100 μg/mL) demonstrated the highest FRAP value (241.5 mM), similar to that of Vit C (256.1 mM), the positive control.

According to Hwang et al. [36], antioxidant activity, as observed in DPPH, ABTS, and FRAP assays, highly correlates with the total phenolic content. Therefore, in the present study, the excellent antioxidant activity of *C. officianalis* indicates a high phenolic content.

### Cell cytotoxicity assay

**Fig. 2:**
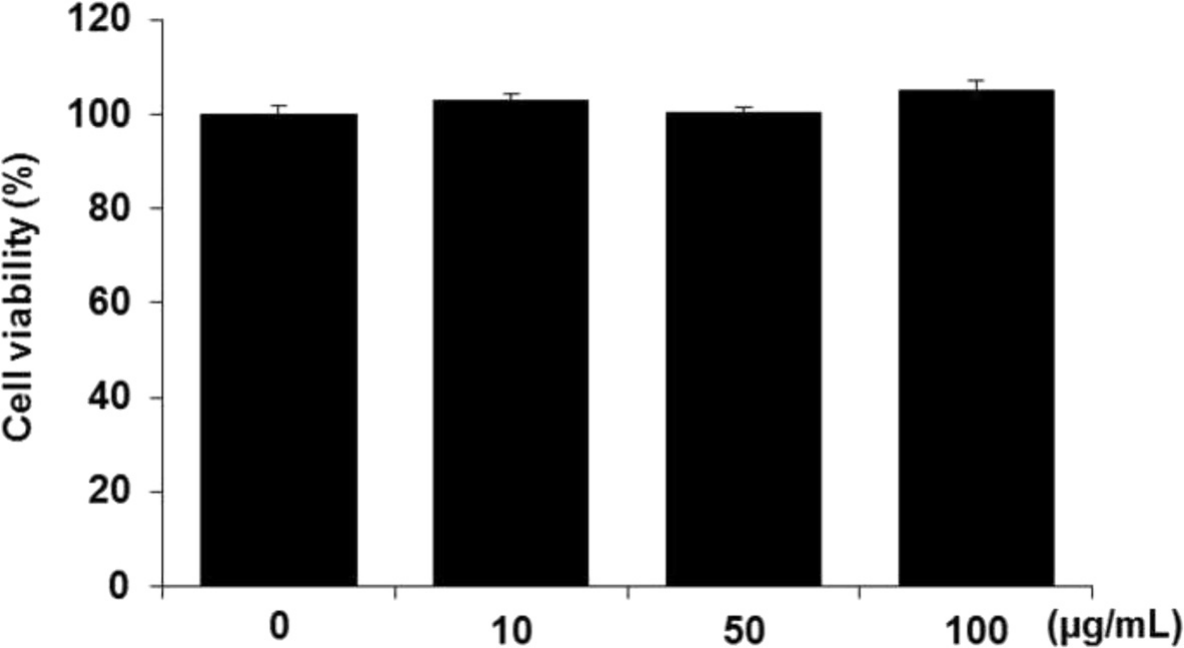
Cytotoxicity effect of *Cornus officinalis* on RAW 264.7 cells. Cells were treated with *Cornus officinalis* at different concentrations (10, 50, and 100 μg/mL). After treatment for 24 h, cell viability was measured with the MTT assay. Values are the mean ± SEM of experiments in triplicate (*n* = 3)

The cytotoxic effects of *C. officianalis* on RAW 264.7 cells were determined by exposing the cells to various concentrations of *C. officianalis* (10, 50, and 100 μg/mL) for 24 h. Our results showed that there was no cytotoxic effect on RAW 264.7 cells at the tested concentration (Fig. 2). Therefore, ethanol extracts of *C. officianalis* with concentrations from 10 to 100 μg/mL were selected for subsequent experiments.

### XO inhibitory activity

XO, an enzyme present in significant concentrations in the gastrointestinal tract and liver, is responsible for the metabolism of hypoxanthine and xanthine to uric acid in the purine catabolic pathway, which produces superoxide radicals. XO is an important biological source of superoxides and has been reported to be present in various pathological processes [37]. In recent years, a number of research groups have investigated potential XO inhibitors from a wide variety of traditional folk medicines.

**Fig. 3:**
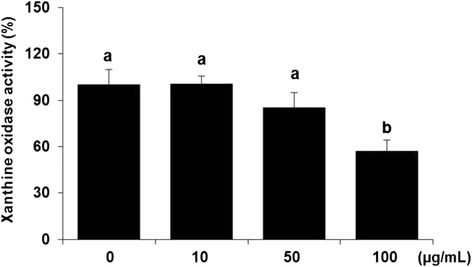
Xanthine oxidase inhibitory effect of various concentrations of *Cornus officinalis* on RAW 264.7 cells. Cells were treated with *Cornus officinalis* at different concentrations (10, 50, and 100 μg/mL). After treatment for 24 h, xanthine oxidase activity was measured with a commercial kit. Values are the mean ± SEM of experiments in triplicate. Values expressed by different letters are significantly different at *p* < 0.05

We confirmed the inhibitory effects of *C. officianalis* ethanol extract on XO activity. As shown in Fig. 3, XO activity decreased in a dose-dependent manner; the inhibitory effects were 100.5 %, 85.0 %, and 57.0 % at 10, 50, and 100 μg/mL of *C. officianalis* ethanol extract, respectively.

In a study by Kim et al. [38], plant polyphenols were found to inhibit enzyme activity by binding to the enzyme-substrate complex instead of directly binding to the active site of the enzyme. Therefore, the XO inhibitory activity observed by *C. officianalis* may be due to polyphenols in *C. officianalis*. Further studies based on the purification and identification of the polyphenol from *C. officianalis* extract is necessary to investigate the exact mechanism of XO inhibitory activity.

### Intracellular reactive oxygen species scavenging activity

**Fig. 4:**
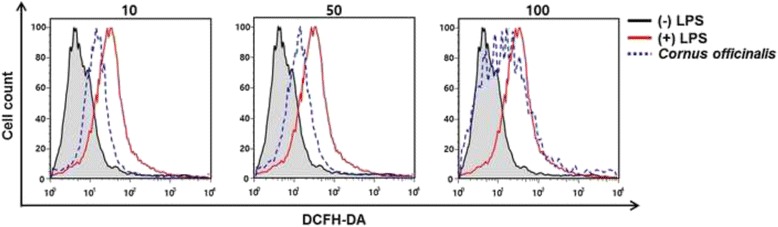
Effects of *Cornus officinalis* on LPS-induced intracellular ROS generation in RAW 264.7 cells. Cells were treated with *Cornus officinalis* extracts at different concentrations (10, 50, and 100 μg/mL). After treatment for 24 h, ROS generation was measured by DCFH-DA staining with flow cytometry analysis

To investigate the intracellular levels of ROS, the cell-permeable probe DCF-DA was utilized. Non-fluorescent DCF-DA, hydrolyzed to DCFH inside the cells, yields highly fluorescent DCF-DA in the presence of intracellular hydrogen peroxide and related peroxides [39]. We examined whether *C. officianalis* extract inhibited LPS-induced ROS generation. As shown in Fig. 4, LPS treatment significantly increased ROS formation in RAW 264.7 cells, as determined by DCF fluorescence. However, treatment with *C. officianalis* extract blocked LPS-induced ROS generation, similar to the results obtained from the antioxidant assays. This evidence suggests that *C. officianalis* extract may prevent the formation of ROS.

### Genes and protein expression of antioxidant enzymes

**Fig. 5:**
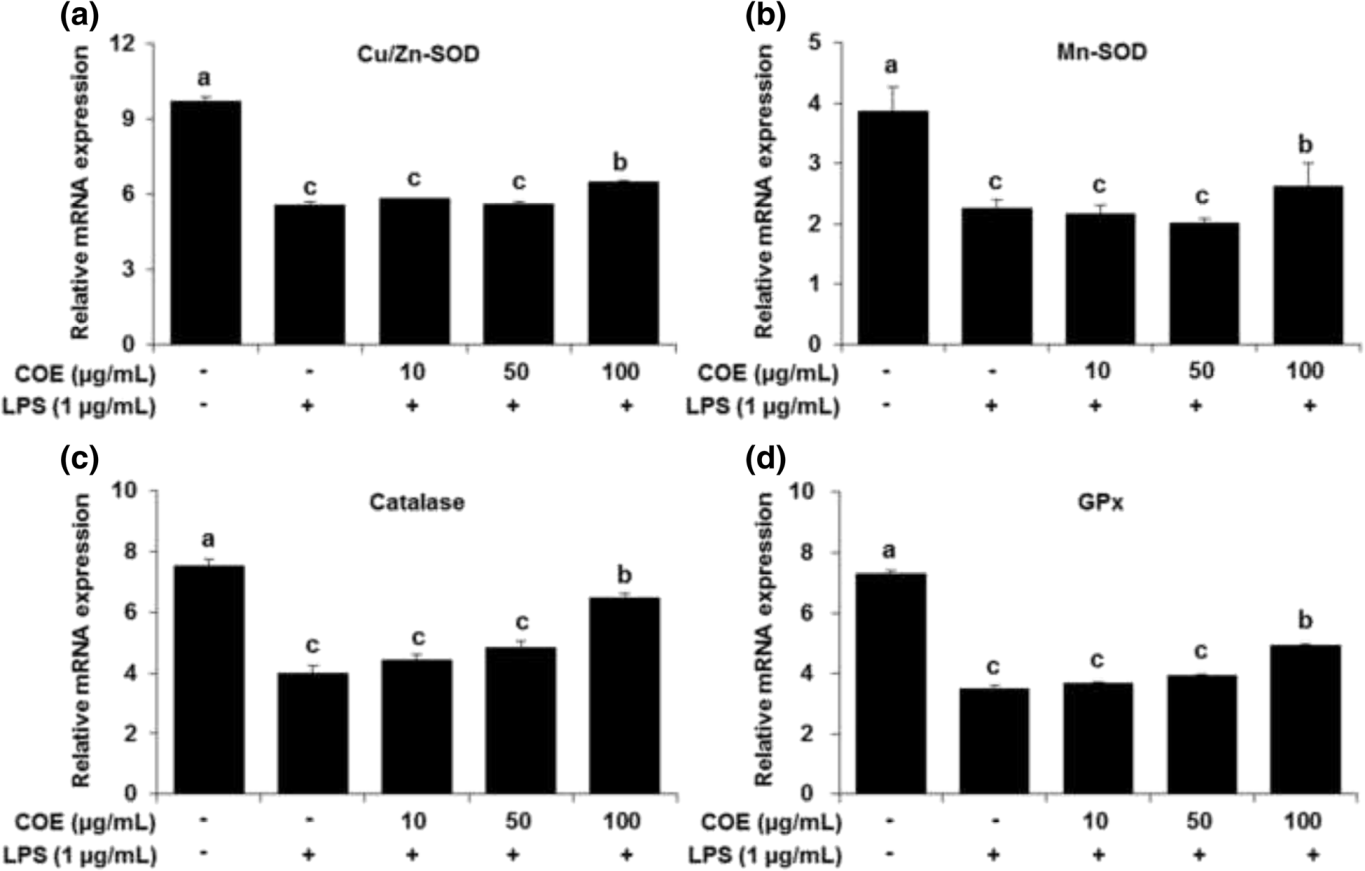
Effect of *Cornus officinalis* extracts on the expression of Cu/Zn-SOD (**a**), Mn-SOD (**b**), catalase (**c**), and GPx (**d**) in RAW 264.7 cells. Cells were treated with *Cornus officinalis* extracts at different concentrations (10, 50, and 100 μg/mL). Values are the mean ± SEM of experiments in triplicate. *p < 0.05 compared to the LPS-treated group. COE; *Cornus officinalis* ethanol extracts

We assessed whether *C. officianalis* ethanol extract affected the expression of genes associated with the antioxidative system, such as Cu/Zn-SOD, Mn-SOD, catalase, and GPx genes, in RAW 264.7 cells. Our data show that *C. officianalis* ethanol extract significantly increased the expression of Cu/Zn-SOD, Mn-SOD, catalase, and GPx compared to that in the LPS-induced control group at 100 μg/mL (Fig. 5). To rule out general defects in oxidative stress in RAW 264.7 cells, the cells were treated with H_2_O_2_. The increased oxidative stress is related to an overproduction of free radicals or deficiency in the antioxidant defense system. The mRNA expression of antioxidant-related enzymes was reduced. However, the *C. officianalis-*treated group showed an increase in the antioxidant-related enzyme mRNA levels (Additional file 1: Figure S1). In addition, we confirmed that *C. officianalis* ethanol extract was indeed responsible for the antioxidant activity in H_2_O_2_-induced RAW 264.7 cells by analyzing Cu/Zn-SOD, MnSOD, catalase, and GPx expression by immunoblotting. Similar to the gene expression results, the antioxidant enzyme levels increased in the *C. officianalis*-treated cells (Additional file 1: Figure S2).

The SODs convert superoxide radicals into hydrogen peroxide and molecular oxygen (O_2_), while catalase and GPx convert hydrogen peroxide into water, and in the case of catalase, to oxygen and water. During homeostasis, SOD sufficiently inactivates superoxides. However, during pathological states such as oxidative stress and diseases, increased levels of superoxides are not inactivated by SOD in the cells and can result in ROS-induced damage. There are three SOD enzymes. MnSOD is localized in the mitochondria, Cu/ZnSOD is located in the cytoplasm and nucleus, and ECSOD is expressed extracellularly in some tissues. Other antioxidant enzymes include catalase, which is found in peroxisomes and cytoplasm, and GPx, which can be found in many sub-cellular components including the mitochondria and nucleus [40–42]. In our results, the *C. officianalis* ethanol extract suppressed oxidative stress by increasing antioxidant enzyme activities including those of SOD, catalase, and GPx. Thus, *C. officianalis* ethanol extract may have a strong antioxidant effect due to *C. officianalis*-induced activation of antioxidative enzymes at different locations within the cells.

These antioxidant enzyme activities are positively correlated with antioxidant effects. The main function of antioxidant enzymes is to neutralize free radicals. Antioxidant enzymes may interrupt an oxidizing chain reaction to minimize the damage caused by free radicals. The risk of acquiring free-radical-related diseases could be reduced by decreasing exposure to free radicals and increasing the intake of antioxidants and antioxidant enzyme-rich foods. Many studies have shown a strong positive linear correlation between antioxidant capacity and antioxidant enzymes of medicinal herbs and other dietary plants. Moreover, these results have also suggested that antioxidant enzyme activity is responsible for their antioxidant capacity [43–45]. In the present study, our data showed that *C. officianalis* extract has potent free radical-scavenging capacities for DPPH and ABTS radicals. Antioxidant capacities in *C. officianalis* were highly correlated with their antioxidant enzyme activities. High activity of SOD, catalase, and GPx increased the free radical-scavenging capacity of the *C. officianalis-*treated group. This study has shown that *C. officianalis* ethanol extract had significant antioxidant activity and increased antioxidant enzyme activity. Thus, we suggest that consuming *C. officianalis* fruit may be beneficial to human health.

## Conclusion

The present study found that *C. officianalis* extract has strong antioxidant activity in cells via activation of the antioxidative enzyme system. Further studies of the mechanism of action of these compounds are underway. Based on these results, *C. officianalis* appears to be a good natural antioxidant agent and could be of significance in the food industry for treating various human and animal diseases.

## Abbreviations

ABTS, 2,2’-azinibis 3-ethyl benzothiazoline-6-sulfonic acid; DCFH-DA, dichlorofluorescein diacetate; DMEM, Dulbecco’s modified Eagle’s medium; DPPH, 1,1-diphenyl-1-picrylhydrazyl; FBS, fetal bovine serum; FeSO_4_, sulfate heptahydrate; GAE, gallic acid equivalent; GAPDH, glyceraldehyde 3-phosphate dehydrogenase; GPx, glutathione peroxidase; LPS, lipopolysaccharides; MTT, 3-(4,5-dimethylthiazol-2-yl)-2,5-diphenyltetrazolium bromide; P/S, penicillin-streptomycin; QE, quercetin equivalent; ROS, reactive oxygen species; RT-PCR, real-time reverse transcription polymerase chain reaction; SEM, standard error of the mean; SOD, superoxide dismutase; TPTZ, 2,4,6-tris(2-pyridyl)-s-triazine; XO, xanthine oxidase.

## Additional file

Additional file 1: Figure S1. Effect of *Cornus officinalis* extracts on the expression of Cu/Zn-SOD (a), Mn-SOD (b), Catalase (c), and GPx (d) genes in RAW 264.7 cells. Cells were treated with H2O2 (100 μM) and *Cornus officinalis* extracts at different concentrations (10, 50, and 100 μg/mL). Values are the mean ± SEM of experiments in triplicate. Values expressed by different letters are significantly different at *p* < 0.05. COE; *Cornus officinalis* ethanol extracts. **Figure S2.** Effect of *Cornus officinalis* extracts on the protein expression of antioxidant enzymes in RAW 264.7 cells. Cells were treated with *Cornus officinalis* extracts at different concentrations (10, 50, and 100 μg/mL). (PPTX 148 kb)
