# Supplementary material for: Antioxidant activities and oxidative stress inhibitory effects of ethanol extracts from Cornus officinalis on raw 264.7 cells
Source: BMC Complement Altern Med. 2016 Jul 8;16:196. doi: 10.1186/s12906-016-1172-3 (PMC4939040; doi:10.1186/s12906-016-1172-3)
Supplement: Additional file 1: Figure S1. — Effect of Cornus officinalis extracts on the expression of Cu/Zn-SOD (a), Mn-SOD (b), Catalase (c), and GPx (d) genes in RAW 264.7 cells. Cells were treated with H2O2 (100 μM) and Cornus officinalis extracts at different concentrations (10, 50, and 100 μg/mL). Values are the mean ± SEM of experiments in triplicate. Values expressed by different letters are significantly different at p < 0.05. COE; Cornus officinalis ethanol extracts. Figure S2. Effect of Cornus officinalis extracts on the protein expression of antioxidant enzymes in RAW 264.7 cells. Cells were treated with Cornus officinalis extracts at different concentrations (10, 50, and 100 μg/mL). (PPTX 148 kb) [file 12906_2016_1172_MOESM1_ESM.pptx]

## Slide 1
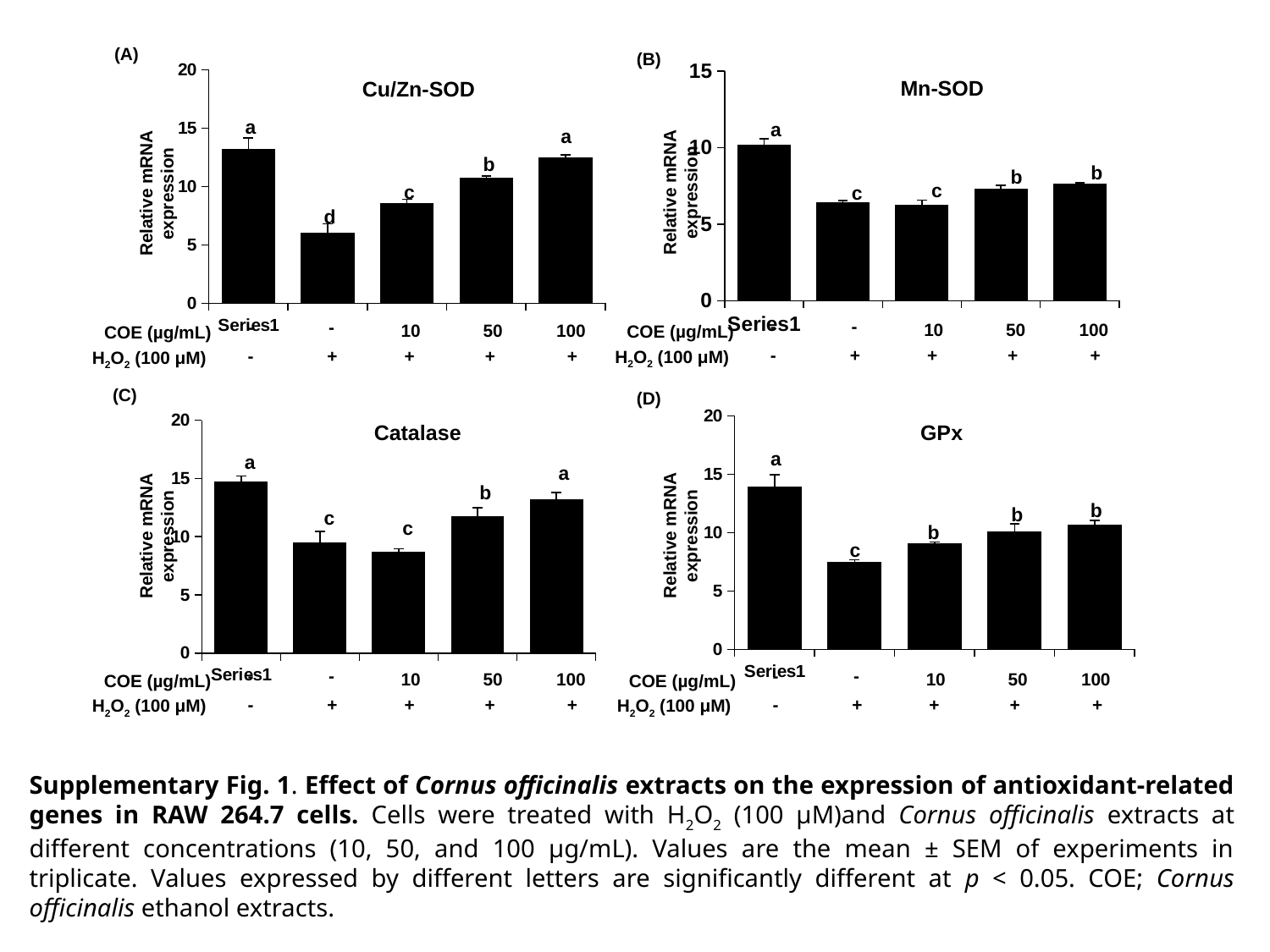

(A)
(B)
### Chart
| Category | |
|---|---|
| | 10.148419727887246 |
| | 6.388389622679525 |
| | 6.212357626994049 |
| | 7.298820793056346 |
| | 7.608802568418009 |
### Chart
| Category | |
|---|---|
| | 13.192679108854014 |
| | 5.98434237967993 |
| | 8.536189763111592 |
| | 10.71105009274106 |
| | 12.445220308144854 |Mn-SOD
Cu/Zn-SOD
Relative mRNA expression
Relative mRNA expression
a
a
a
b
b
*
b
c
c
c
d
-
-
10
50
100
COE (µg/mL)
-
+
+
+
+
H2O2 (100 μM)
-
-
10
50
100
COE (µg/mL)
-
+
+
+
+
H2O2 (100 μM)
(C)
(D)
### Chart
| Category | |
|---|---|
| | 13.899241738497851 |
| | 7.45698445125111 |
| | 9.013692389098724 |
| | 10.038568257048473 |
| | 10.6631308192598 |
### Chart
| Category | |
|---|---|
| | 14.727335462191917 |
| | 9.45913155334467 |
| | 8.709195657233103 |
| | 11.722703516699141 |
| | 13.164202146948279 |Catalase
GPx
Relative mRNA expression
Relative mRNA expression
a
a
a
b
b
b
c
c
b
c
-
-
10
50
100
COE (µg/mL)
-
+
+
+
+
H2O2 (100 μM)
-
-
10
50
100
COE (µg/mL)
-
+
+
+
+
H2O2 (100 μM)
Supplementary Fig. 1. Effect of Cornus officinalis extracts on the expression of antioxidant-related genes in RAW 264.7 cells. Cells were treated with H2O2 (100 μM)and Cornus officinalis extracts at different concentrations (10, 50, and 100 μg/mL). Values are the mean ± SEM of experiments in triplicate. Values expressed by different letters are significantly different at p < 0.05. COE; Cornus officinalis ethanol extracts.

## Slide 2
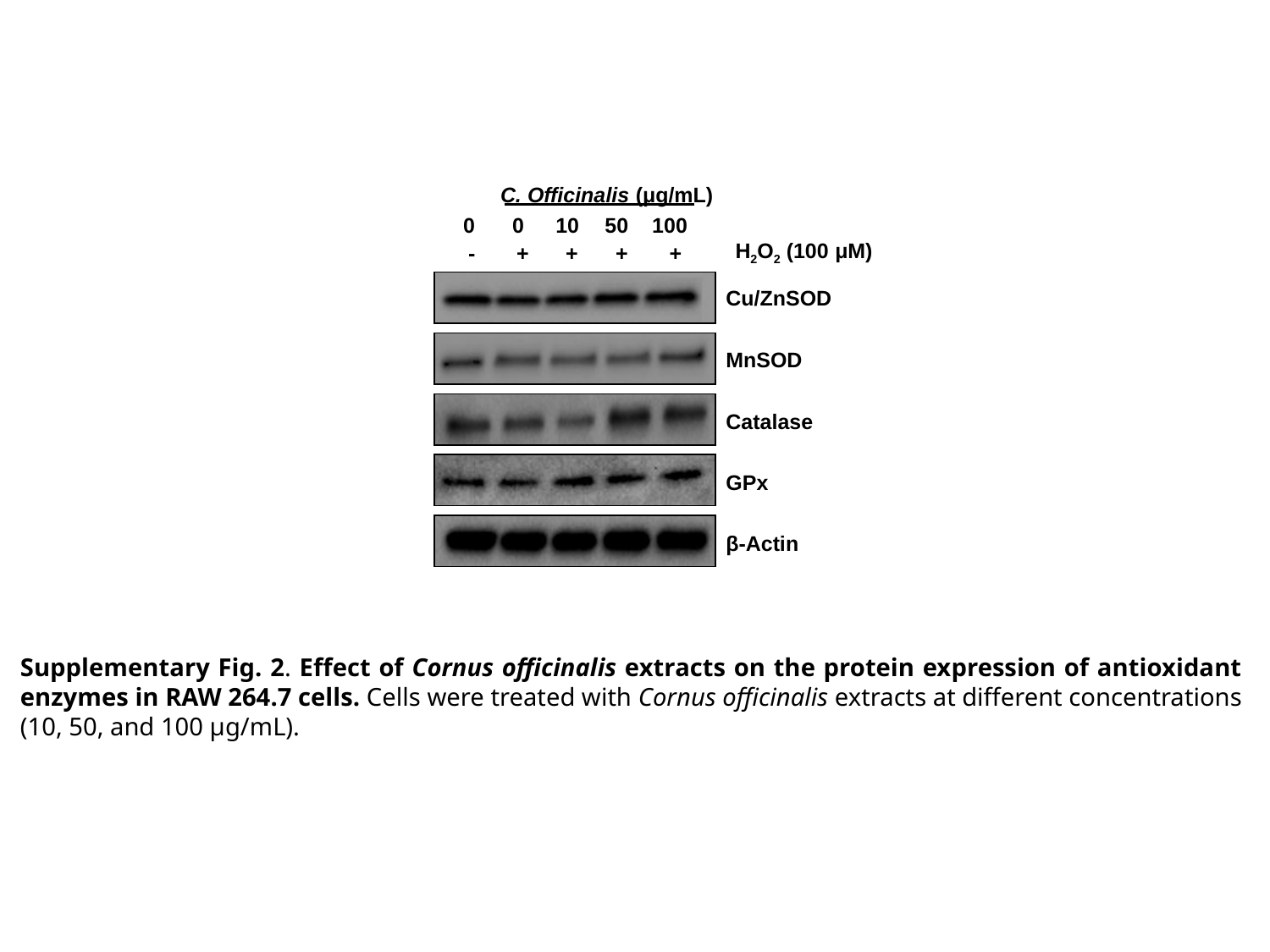

C. Officinalis (μg/mL)
0
0
10
50
100
-
+
+
+
+
H2O2 (100 μM)
Cu/ZnSOD
MnSOD
Catalase
GPx
β-Actin
Supplementary Fig. 2. Effect of Cornus officinalis extracts on the protein expression of antioxidant enzymes in RAW 264.7 cells. Cells were treated with Cornus officinalis extracts at different concentrations (10, 50, and 100 μg/mL).
